# Supplementary material for: Antimicrobial Activity of Protein Fraction from Naja ashei Venom against Staphylococcus epidermidis
Source: Molecules. 2020 Jan 10;25(2):293. doi: 10.3390/molecules25020293 (PMC7024148; doi:10.3390/molecules25020293)
Supplement: Supplementary file 1 [file molecules-25-00293-s001.zip › Table S2.docx]

**Table S2.** Identified proteins from Ig-like domain-containing protein group. (+) denotes fractions in which a given protein was detected, (-) denotes fractions in which the presence of a given protein was not observed.

| **Accession number** | **Protein name** | **Species** | **Source** | **SUPFAM [59]** | **Fractions** | | | | | | | | |
| --- | --- | --- | --- | --- | --- | --- | --- | --- | --- | --- | --- | --- | --- |
|  |  |  |  |  | **F1** | **F2** | **F3** | **F4** | **F5** | **F6** | **F7** | **F8** | **F10** |
| A0A2D4EV48 | Ig-like domain-containing protein (Fragment) (A0A2D4EV48_MICCO) | *Micrurus corallinus* | mRNA | SSF48726 | - | + | - | - | - | - | - | - | - |
| A0A2D4PVS5 | Uncharacterized protein (Fragment) (A0A2D4PVS5_MICSU) | *Micrurus surinamensis* | mRNA | SSF48726 | + | - | - | - | - | - | - | - | - |
| A0A2D4M1A3 | Ig-like domain-containing protein (Fragment) (A0A2D4M1A3_9SAUR) | *Micrurus spixii* | mRNA | SSF48726 | + | + | - | - | - | - | - | - | - |
| A0A2D4LEH0 | Uncharacterized protein (Fragment) (A0A2D4LEH0_9SAUR) | *Micrurus spixii* | mRNA | SSF48726 | + | + | + | - | - | - | - | - | - |
| A0A2D4LE86 | Uncharacterized protein (Fragment) (A0A2D4LE86_9SAUR) | *Micrurus spixii* | mRNA | SSF48726 | + | + | - | - | - | - | - | - | - |
| A0A0B8RRE3 | Ig lambda chain (A0A0B8RRE3_BOIIR) | *Boiga irregularis* | mRNA | SSF48726 | + | + | - | - | - | - | - | - | - |
| V8N9M9 | Uncharacterized protein (Fragment) (V8N9M9_OPHHA) | *Ophiophagus hannah* | DNA | SSF48726 | - | + | - | - | - | - | - | - | - |
| V8P9R0 | Uncharacterized protein (V8P9R0_OPHHA) | *Ophiophagus hannah* | DNA | SSF48726 | + | + | + | - | - | - | - | - | + |
| A0A2D4HAH3 | IGv domain-containing protein (Fragment) (A0A2D4HAH3_MICLE) | *Micruruslemniscatuslemniscatus* | mRNA | SSF48726 | - | + | - | - | - | - | - | - | - |
| A0A2D4LP04 | Ig-like domain-containing protein (Fragment) (A0A2D4LP04_9SAUR) | *Micrurusspixii* | mRNA | SSF48726  SSF54452 | + | - | - | - | - | - | - | - | - |
| V8NJK5 | Uncharacterized protein (Fragment) (V8NJK5_OPHHA) | *Ophiophagus hannah* | DNA | SSF48726 | + | - | - | - | - | - | - | - | - |
| A0A2D4HEV1 | IGv domain-containing protein (Fragment) (A0A2D4HEV1_MICLE) | *Micrurus lemniscatus lemniscatus* | mRNA | SSF48726 | + | + | - | - | - | - | - | - | - |
| A0A2D4HR81 | Ig-like domain-containing protein (Fragment) (A0A2D4HR81_MICLE) | *Micrurus lemniscatus lemniscatus* | mRNA | SSF48726 | + | + | - | - | - | - | - | - | - |
| V8NFA2 | Ig-like domain-containing protein (Fragment) (V8NFA2_OPHHA) | *Ophiophagus hannah* | DNA | SSF48726  SSF54452 | + | - | - | - | - | - | - | - | + |
| A0A0B8RUH1 | Ig lambda chain (A0A0B8RUH1_BOIIR) | *Boiga irregularis* | mRNA | SSF48726 | - | + | - | - | - | - | - | - | - |
| A0A2D4EU10 | Uncharacterized protein (Fragment) (A0A2D4EU10_MICCO) | *Micrurus corallinus* | mRNA | SSF48726 | - | + | + | - | - | - | - | - | + |
| A0A2D4HEU7 | IGv domain-containing protein (Fragment) (A0A2D4HEU7_MICLE) | *Micrurus lemniscatus lemniscatus* | mRNA | SSF48726 | - | + | - | - | - | - | - | - | - |
| A0A2D4PVU9 | IGv domain-containing protein (Fragment) (A0A2D4PVU9_MICSU) | *Micrurus surinamensis* | mRNA | SSF48726 | - | + | - | - | - | - | - | - | - |
| A0A2D4LDN5 | Uncharacterized protein (Fragment) (A0A2D4LDN5_9SAUR) | *Micrurus spixii* | mRNA | SSF48726 | - | + | - | - | - | - | - | - | - |
| A0A2D4PIZ6 | Uncharacterized protein (Fragment) (A0A2D4PIZ6_MICSU) | *Micrurus surinamensis* | mRNA | SSF48726 | - | + | - | - | - | - | - | - | - |
| V8N590 | Uncharacterized protein (Fragment) (V8N590_OPHHA) | *Ophiophagushannah* | DNA | SSF48726 | - | + | - | - | - | - | - | - | - |
| V8NBD1 | Pregnancy-specific beta-1-glycoprotein 5 (V8NBD1_OPHHA) | *Ophiophagus hannah* | DNA | SSF48726 | - | + | + | - | - | - | - | - | + |
| A0A2D4LE23 | Uncharacterized protein (Fragment) (A0A2D4LE23_9SAUR) | *Micrurus spixii* | mRNA | SSF48726 | - | + | - | - | - | - | - | - | - |
