# Supplementary material for: Antimicrobial Activity of Protein Fraction from Naja ashei Venom against Staphylococcus epidermidis
Source: Molecules. 2020 Jan 10;25(2):293. doi: 10.3390/molecules25020293 (PMC7024148; doi:10.3390/molecules25020293)
Supplement: Supplementary file 1 [file molecules-25-00293-s001.zip › Table S3.docx]

**Table S3.** The percentage share of the Ig-like domain-containing protein class in individual fractions and total collected material.

| **Fraction** | **% of total collected material** | **% of Ig-like group** | **% of Ig-like group in total collected material** |
| --- | --- | --- | --- |
| 1 | 1 | 18 | 0.18 |
| 2 | 4 | 13 | 0.52 |
| 3 | 12 | 1 | 0.12 |
| 4 | 25 | 0 | 0 |
| 5 | 15 | 0 | 0 |
| 6 | 15 | 0 | 0 |
| 7 | 8 | 0 | 0 |
| 8 | 16 | 0 | 0 |
| 9 | 3 | 0 | 0 |
| 10 | 1 | 1.4 | 0.014 |
| total | 100 | - | 0.834 |
